# Supplementary material for: COVID-19 pandemic in Saint Petersburg, Russia: Combining population-based serological study and surveillance data
Source: PLoS One. 2022 Jun 15;17(6):e0266945. doi: 10.1371/journal.pone.0266945 (PMC9200332; doi:10.1371/journal.pone.0266945)

# Supplementary materials

## COVID-19 pandemic in Saint Petersburg, Russia: combining surveillance and population-based serological study data in May, 2020–April, 2021

Anton Barchuk, Dmitriy Skougarevskiy, Alexei Kouprianov, Daniil Shirokov, Olga Dudkina, Rustam Tursun-zade, Mariia Sergeeva, Varvara Tychkova, Andrey Komissarov, Alena Zheltukhina, Dmitry Lioznov, Artur Isaev, Ekaterina Pomerantseva, Svetlana Zhikrivetskaya, Yana Sofronova, Konstantin Blagodatskikh, Kirill Titaev, Lubov Barabanova, Daria Danilenko

### Data appendix

The federal government and St. Petersburg city government made most of the detailed statistics regarding COVID-19 available. However the data are scattered across different sources. The number of daily new cases was obtained from the official federal government website (`stopcoronavirus.rf`, https://xn--80aesfpebagmfblc0a.xn--p1ai/). The daily reports of new cases were also provided by the St. Petersburg city government and the city Health Committee. While the official website provided a somewhat smoothed pandemic curve, the St. Petersburg city government and Health Committee data looked closer to sources from other countries, with seasonal fluctuations on weekends and holidays. However, the city data are available only from early December 2020. The number of COVID-19 deaths was obtained from the official data from `stopcoronavirus.rf`. The number of excess deaths was obtained from Kobak (2020), the calculation was based on monthly data from the Federal State Statistics Service of Russia on deaths from any causes [11]. This data is constructed by subtracting the linear trend in monthly deaths over 2015–19 from the monthly deaths data in 2020–21 and is available online https://github.com/dkobak/excess-mortality. We obtained the number of tests to detect SARS-CoV-2 from the official St. Petersburg city government Telegram channel https://t.me/koronavirusspb. The number of hospital admissions are extracted from the St. Petersburg city government and Health Committee reports. The ongoing surveillance for SARS-CoV-2 VOCs in St. Petersburg is carried out by the Smorodintsev Research Institute of Influenza and is described in detail elsewhere [18].

### Statistical appendix: estimating the IR and IFR with the Bayesian evidence synthesis model

**Observables** We conduct $K$ cross-sections of serosurvey of adult population of St. Petersburg, Russia. In each cross-section $k = 1, \ldots, K$ we randomly select $T_k$ individuals to get tested out of $P_k$ individuals at risk of infection in the city. Out of those tested we identify $CC_k$ seropositive individuals with confirmed cases of SARS-CoV-2. In each cross-section $k$ we also observe the cumulative number of deaths $D_k$ attributed to COVID-19 since the pandemic onset in the city.

**Latent variables** We need to make inference on following variables:

- $C_k$ — the cumulative total number of infected individuals by wave $k$,
- $IR_k$ — the true infection rate (proportion of population which has been infected by cross-section $k$), which is the expected value $\mathrm{E}\left[{}^{C_k}/_{P_k}\right]$,
- $IFR_k$ — the true underlying infection fatality rate, which is the expected value $\mathrm{E}\left[{}^{D_k}/_{C_k}|C_k\right]$.

To estimate the IFR across the study cross-sections we closely follow [20] who proposed a simple framework for Bayesian evidence synthesis.

**Distributional and modeling assumptions** We make the following assumptions on the distribution of the latent variables:

$$CC_k \sim \text{Binomial}\left(T_k, {}^{C_k}/{}_{P_k}\right),$$
$$C_k \sim \text{Binomial}\left(P_k, IR_k\right),$$
$$D_k | C_k \sim \text{Binomial}\left(C_k, IFR_k\right).$$

Following [20], to improve the MCMC mixing we replace the assumption for $CC_k$ with $CC_k \sim \text{Binomial}\left(T_k, IFR_k\right)$. Then we can replace the conditional assumption $D_k|C_k$ with the unconditional

$$D_k \sim \text{Binomial}\left(P_k, IFR_k \times IR_k\right).$$

Next we assume that per-cross-section $IFR_k$ and $IR_k$ are distributed according to a random effects model:

$$g\left(IFR_k\right) \sim \mathcal{N}\left(\theta, \tau^2\right),$$
$$g\left(IR_k\right) \sim \mathcal{N}\left(\beta, \sigma^2\right),$$

where $g\left(\bullet\right) \equiv \log\left(-\log\left(1-\bullet\right)\right)$ is the complimentary log-log link function, $\theta$ is the mean clog-log-transformed infection fatality rate across the study cross-sections, $\tau$ reflects the variability in between-cross-section IFR estimates, $\beta$ is the mean clog-log-transformed infection rate across the cross-sections, $\sigma$ captures the variability in between-cross-section IR estimates.

**Prior elicitation** Following [20, 26] we consider two sets of priors on model parameters:

- Weakly informative priors: $g^{-1}\left(\theta\right) \sim \text{Beta}\left(0.3, 30\right)$, $g^{-1}\left(\beta\right) \sim \text{Beta}\left(1, 30\right)$, $\sigma \sim \text{half-}\mathcal{N}\left(0, 10\right)$, $\tau \sim \text{half-}\mathcal{N}\left(0, 10\right)$;
- Non-informative flat priors: $g^{-1}\left(\theta\right) \sim \text{Uniform}\left(0, 1\right)$, $g^{-1}\left(\beta\right) \sim \text{Uniform}\left(0, 1\right)$, $\sigma \sim \text{half-}\mathcal{N}\left(0, 100\right)$, $\tau \sim \text{half-}\mathcal{N}\left(0, 100\right)$.

**Estimation and inference** The model is fit with JAGS [27] with 5 independent chains, each with 2 million draws (20% burn-in, thinning of 100). We then report the median estimates of per-cross-section $IFR_k$ and $IR_k$ and their 95% highest probability density credible intervals [28].

**Data** To fit the model we need to acknowledge multiple data constraints. For $P_k$ we assume that the entire adult ($\geq 18$ years old) population of the city is at risk of infection (in a sensitivity analysis we consider the entire city population instead). For cross-sections one and two we take the adult city population count as of January 1, 2020 from the Federal State Statistics Service of Russia*, 4 451 025 individuals. The data on the adult population as of January 1, 2021 is not available at the time of writing of this paper. However, the official data on the total city population is available† and amounts to 5 384 342 people as of January 1,

*https://gks.ru/bgd/regl/b20_111/Main.htm
†https://petrostat.gks.ru/folder/27595

2021 and 5 398 064 as of January 1, 2020. We assume that the adult population followed the same trend as the total population (a -0.25% decline) in 2020 and assume $P_k = 4\ 451\ 025 \times (1 - 0 \cdot 0025) = 4\ 439\ 897$ individuals for cross-sections three and four.

We do not take the values $T_k$ and $CC_k$ directly from the per-cross-section test data. To arrive at the seroprevalence estimate in our study we adjusted those naïve figures for test performance and non-response bias. Instead of using the raw counts we invert the reported 95% CI for the seroprevalence estimate for cross-section $k$. Using a beta prior on the probability of success for a binomial distribution, we can determine a two-sided confidence interval from a beta posterior for any given $T_k$ and $CC_k$. We define the values of $T_k$ and $CC_k$ that correspond to the reported seroprevalence 95% CI for ELISA Coronapass from Table 1 adjusted for non-response and test characteristics as the effective $T_k^{effective}$ and $CC_k^{effective}$ and use those values in the model. Such "inverting uncertainty intervals" approach of [20] allows us to easily incorporate our seroprevalence adjustments coming from a frequentist univariate imputation model into a Bayesian evidence synthesis model.

When it comes to the cumulative number of deaths $D_k$ by cross-section $k$ an obvious question is what date to use to compute this figure for each study cross-section. [29] suggest compute the total number of deaths up until seven days after the cross-section mid-point. [20] propose to treat $D_k$ as an interval censored variable where we do not know its true value but observe its lower and upper bounds $D_k^{lower}$ and $D_k^{upper}$ for each cross-section. The authors define $D_k^{lower/upper}$ as the total number of deaths from the pandemic onset until 14 days after the start/the end of the cross-section $k$, respectively. We adopt this approach as it allows for uncertainty in the actual death counts.

Another concern is reliability of the reported deaths data. We use two sources for $D_k^{lower/upper}$. The first is the official national government website (`stopcoronavirus.rf`) that provides daily data on COVID-related deaths in St. Petersburg. The second is excess deaths estimation based on monthly data from the Federal State Statistics Service of Russia [11]. We find it valuable to compute the IFR and IR using the data from both sources given the voiced concerns about under-reporting of COVID-related deaths in the country. For monthly excess deaths data we consider the cumulative excess deaths from January 1, 2020 to the month of the cross-section start to define $D_k^{lower}$ and the cumulative excess deaths from January 1, 2020 to the month of the cross-section end to define $D_k^{upper}$. All the variables used in the estimation are reported in Table S1.

**Table S1.** Data used for IR/IFR estimation in the Bayesian evidence synthesis model

| $k$ (cross-section dates) | $P_k^{adult}$ | $P_k^{all}$ | $T_k^{effective}$ | $CC_k^{effective}$ | $D_{k,official}^{lower}$ | $D_{k,official}^{upper}$ | $D_{k,excess}^{lower}$ | $D_{k,excess}^{upper}$ |
|---|---|---|---|---|---|---|---|---|
| 1 (2020-05-25 – 2020-06-28) | 4 451 025 | 5 398 064 | 827 | 79 | 392 | 1603 | 2978 | 4776 |
| 2 (2020-07-20 – 2020-08-08) | 4 451 025 | 5 398 064 | 385 | 50 | 2062 | 2421 | 5949 | 6537 |
| 3 (2020-10-12 – 2020-12-06) | 4 439 897 | 5 384 342 | 999 | 228 | 3627 | 6840 | 9045 | 14468 |
| 4 (2020-02-15 – 2020-04-04) | 4 439 897 | 5 384 342 | 550 | 241 | 11112 | 12811 | 18944 | 21426 |

**Results** The per-cross-section estimates of IR/IFR under different priors and death intervals are reported in Table S2.

**Per-age and sex IR and IFR** Our approach can be easily applied to another problem. Suppose now that $k$ indexes sex and age groups within one serosurvey cross-section. Then we can use the same logic to estimate IR and IFR for each age group-sex combinations.

We predict ELISA Coronapass-based seroprevalence within each sex and age group combination from our baseline univariate

**Table S2.** Estimated IR/IFR across the study cross-sections from the Bayesian evidence synthesis model

| population | only adult population | | | | | | | | all population | |
|---|---|---|---|---|---|---|---|---|---|---|
| priors | weakly informative | | | | non-informative | | | | weakly inform. | |
| estimate | IR | | IFR | | IR | | IFR | | IR | IFR |
| deaths | official | excess | official | excess | official | excess | official | excess | excess | |
| 1 | 9.28 | 9.22 | 0.29 | 1.01 | 9.41 | 9.30 | 0.27 | 1.01 | 9.22 | 0.83 |
| | (7.26–11.26) | (7.54–10.97) | (0.10–0.42) | (0.75–1.22) | (7.45–11.45) | (7.53–11.02) | (0.09–0.41) | (0.74–1.21) | (7.50–10.94) | (0.62–1.00) |
| 2 | 12.73 | 13.30 | 0.40 | 1.05 | 12.91 | 13.35 | 0.39 | 1.05 | 13.28 | 0.87 |
| | (9.64–16.03) | (10.72–15.72) | (0.29–0.51) | (0.87–1.28) | (9.80–16.33) | (10.81–15.83) | (0.29–0.51) | (0.86–1.27) | (10.73–15.75) | (0.71–1.05) |
| 3 | 22.78 | 22.84 | 0.48 | 1.05 | 22.82 | 22.87 | 0.48 | 1.06 | 22.84 | 0.87 |
| | (20.26–25.41) | (20.41–25.39) | (0.34–0.66) | (0.87–1.33) | (20.25–25.40) | (20.42–25.43) | (0.34–0.67) | (0.87–1.34) | (20.40–25.41) | (0.72–1.10) |
| 4 | 43.84 | 43.64 | 0.61 | 1.04 | 43.80 | 43.65 | 0.61 | 1.04 | 43.64 | 0.86 |
| | (39.85–48.09) | (39.75–47.53) | (0.54–0.69) | (0.93–1.16) | (39.63–47.83) | (39.63–47.54) | (0.54–0.69) | (0.93–1.16) | (39.68–47.47) | (0.77–0.96) |
| Overall | 8.69 | 8.74 | 0.43 | 1.04 | 23.38 | 23.29 | 0.50 | 1.04 | 8.79 | 0.86 |
| | (0.85–17.77) | (1.05–18.09) | (0.11–0.82) | (0.80–1.31) | (4.25–63.35) | (4.53–63.94) | (0.04–19.63) | (0.80–1.35) | (0.91–18.07) | (0.66–1.08) |

model for cross-section 4 where we use an interaction between individual age group and sex instead of treating them as linearly separable variables (as reported in Table S8) and define more fine-grained age groups. Then we invert the estimated CI for the seroprevalence to compute the $T_k^{effective}$ and $CC_k^{effective}$ (see Table S3). For per-group population $P_k$ we use data as of the beginning of 2020 since no data for 2021 is available yet.

When it comes to $D_k^{lower}$ and $D_k^{upper}$ we need to acknowledge that, to the best of our knowledge, no official data on deaths from COVID-19 disaggregated by age and sex exists. For this reason, we rely on excess deaths data estimation. We gather official yearly data on deaths in 2016–19 by age group and sex and quarterly data on deaths in 2020–21 to compute our $D_{k,excess}^{lower/upper}$. We used quarterly data of age and sex-specific number of deaths and population from the Federal State Statistics Service of Russia. First, we combined the number of deaths from all causes from the second, third, and fourth quarter of 2020, and the first quarter of 2021 (the pandemic year). We treated the pandemic year as a calendar year, as it captures all seasonal trends, and includes all periods when excess deaths due to COVID-19 are expected. The first case of SARS-CoV-2 infection was registered in Saint Petersburg in Russia on March 5, 2020, and it is not likely that the first quarter of 2020 contributed to excess mortality due to COVID-19. We estimated expected deaths by using a Poisson model that accounts for annual temporal trends within each age and sex-specific group with an offset that accounts for the population size in each group. For each age and sex-specific group, the model used mortality data for 2016-19 to estimate the expected number of deaths in each group for 2020. Then the predicted lower and upper bound for expected death count in 2020 was used to estimate the number of excess death in the pandemic year. The cumulative number of deaths across all age and sex-specific groups combined (the lower bound was 18 631 and the upper was 22 289) was in line with the excess deaths estimation based on monthly data from the Federal State Statistics Service of Russia (the lower bound was 18 944 and the upper 21 426) [11].

The estimated IR and IFR for each age and sex-specific group combination are in Table S4.

**Table S3.** Data used for IR/IFR estimation for cross-section 4 (ELISA Coronapass) in the Bayesian evidence synthesis model by age group and sex

| Sex | Age group | $P_k^{group}$ | Seroprevalence | $T_k^{effective}$ | $CC_k^{effective}$ | $D_{k,excess}^{lower}$ | $D_{k,excess}^{upper}$ |
|---|---|---|---|---|---|---|---|
| Female | 18–29 | 419 516 | 40.2 (31.3–49.2) | 115 | 46 | 0 | 36 |
| Female | 30–39 | 543 892 | 44.6 (38.2–50.9) | 234 | 104 | 57 | 182 |
| Female | 40–49 | 432 308 | 50.2 (41.7–58.7) | 131 | 66 | 167 | 341 |
| Female | 50–59 | 418 371 | 49.2 (40.4–58.0) | 122 | 60 | 367 | 596 |
| Female | 60–69 | 437 588 | 34.4 (22.0–46.8) | 54 | 18 | 1412 | 1755 |
| Female | 70+ | 482 856 | 37.4 (17.6–57.3) | 20 | 7 | 7679 | 8509 |
| Male | 18–29 | 413 098 | 44.6 (32.8–56.4) | 65 | 29 | 0 | 105 |
| Male | 30–39 | 527 930 | 61.5 (52.4–70.7) | 105 | 65 | 161 | 352 |
| Male | 40–49 | 404 853 | 54.1 (43.1–65.0) | 79 | 43 | 528 | 791 |
| Male | 50–59 | 342 243 | 63.5 (49.9–77.1) | 45 | 29 | 856 | 1167 |
| Male | 60–69 | 281 789 | 56.5 (36.4–76.6) | 21 | 12 | 2111 | 2527 |
| Male | 70+ | 241 605 | 33.6 (1.3–65.9) | 5 | 1 | 5330 | 5928 |

**Table S4.** Estimated IR/IFR across the age and sex groups from the Bayesian evidence synthesis model, cross-section 4, ELISA Coronapass

| priors | weakly informative | | | | non-informative | | | |
|---|---|---|---|---|---|---|---|---|
| estimate | IR | | IFR | | IR | | IFR | |
| sex | female | male | female | male | female | male | female | male |
| 18–29 | 40.04 (31.86–48.73) | 43.54 (32.67–54.11) | 0.01 (0.00–0.02) | 0.02 (0.00–0.06) | 42.32 (34.10–50.11) | 45.84 (36.07–55.33) | 0.01 (0.00–0.02) | 0.02 (0.00–0.05) |
| 30–39 | 44.11 (38.13–50.09) | 58.57 (49.22–68.27) | 0.05 (0.02–0.08) | 0.08 (0.05–0.12) | 45.06 (39.11–50.84) | 57.66 (48.45–67.11) | 0.05 (0.02–0.08) | 0.08 (0.05–0.12) |
| 40–49 | 49.00 (41.32–57.24) | 51.63 (41.64–62.02) | 0.11 (0.07–0.17) | 0.31 (0.22–0.41) | 49.57 (42.32–57.28) | 51.86 (42.88–61.42) | 0.11 (0.07–0.16) | 0.31 (0.22–0.40) |
| 50–59 | 47.96 (39.97–56.22) | 57.75 (44.79–71.93) | 0.23 (0.17–0.32) | 0.51 (0.37–0.67) | 48.72 (41.23–56.51) | 56.39 (45.67–69.94) | 0.23 (0.16–0.31) | 0.52 (0.38–0.68) |
| 60–69 | 36.09 (24.80–47.93) | 49.91 (34.01–67.33) | 1.00 (0.70–1.43) | 1.64 (1.13–2.30) | 40.65 (29.54–50.86) | 51.11 (38.40–66.05) | 0.89 (0.65–1.20) | 1.60 (1.17–2.09) |
| 70+ | 39.22 (22.50–53.98) | 38.37 (15.91–57.49) | 4.27 (2.76–6.73) | 6.06 (3.47–12.37) | 44.68 (30.09–56.77) | 45.91 (28.86–61.22) | 3.75 (2.77–5.23) | 5.07 (3.51–7.49) |

**Table S5.** Summary statistics across study cross-sections

| cross-section dates | 2020-05-25 – 2020-06-28 | | | | 2020-07-20 – 2020-08-08 | | 2020-10-12 – 2020-12-06 | | | | 2020-02-15 – 2020-04-04 | |
|---|---|---|---|---|---|---|---|---|---|---|---|---|
| subsample | interviewed | | tested | | tested | | interviewed | | tested | | tested** | |
| statistic | N | Mean | N | Mean | N | Mean | N | Mean | N | Mean | N | Mean |
| Male | 6,400 | 0.412 | 1,038 | 0.372 | 497 | 0.374 | 7,718 | 0.424 | 1,391 | 0.341 | 1,185 | 0.339 |
| Age group 18–34 | 6,400 | 0.365 | 1,038 | 0.382 | 497 | 0.356 | 7,718 | 0.331 | 1,391 | 0.369 | 1,185 | 0.341 |
| Age group 35–49 | 6,400 | 0.318 | 1,038 | 0.344 | 497 | 0.332 | 7,718 | 0.318 | 1,391 | 0.361 | 1,185 | 0.372 |
| Age group 50–64 | 6,400 | 0.199 | 1,038 | 0.210 | 497 | 0.249 | 7,718 | 0.207 | 1,391 | 0.206 | 1,185 | 0.229 |
| Age group 65+ | 6,400 | 0.119 | 1,038 | 0.065 | 497 | 0.062 | 7,718 | 0.144 | 1,391 | 0.064 | 1,185 | 0.058 |
| Higher education | 6,400 | 0.671 | 1,038 | 0.828 | 497 | 0.853 | 7,718 | 0.619 | 1,391 | 0.797 | 1,185 | 0.824 |
| Higher income | 6,063 | 0.425 | 999 | 0.504 | 480 | 0.533 | 6,930 | 0.365 | 1,303 | 0.424 | 1,118 | 0.442 |
| Respondent lives alone | 6,400 | 0.187 | 1,038 | 0.188 | 497 | 0.217 | 7,718 | 0.191 | 1,391 | 0.171 | 1,185 | 0.169 |
| Started to wash hands more often since pandemic | 6,345 | 0.655 | 1,033 | 0.712 | 493 | 0.728 | 7,637 | 0.634 | 1,388 | 0.705 | 1,180 | 0.709 |
| History of illness in the last 3 months | 6,321 | 0.316 | 1,031 | 0.440 | 496 | 0.435 | 7,185 | 0.564 | 1,337 | 0.690 | 1,143 | 0.721 |
| History of COVID-19 testing | 6,400 | 0.152 | 1,038 | 0.225 | 497 | 0.243 | 7,718 | 0.360 | 1,391 | 0.447 | 1,143 | 0.721 |
| Current smoker* | — | — | 949 | 0.205 | 450 | 0.182 | 7,718 | 0.315 | 1,391 | 0.254 | — | — |
| Encouraged to participate in study | 6,400 | 0.231 | 1,038 | 0.232 | 497 | 1.000 | 7,718 | 0.517 | 1,391 | 0.566 | 1,185 | 0.000 |
| CMIA Abbott positive | — | — | 1,038 | 0.093 | 497 | 0.139 | — | — | 1,390 | 0.164 | — | — |
| ELISA Genetico positive | — | — | 1,035 | 0.103 | 495 | 0.147 | — | — | 1,378 | 0.227 | 1,182 | 0.532 |
| ELISA Vector positive | — | — | — | — | — | — | 1,348 | 0.247 | 1,348 | 0.247 | 1,169 | 0.546 |

* — current smoking status variable is gathered from the paper-based survey of tested individuals in the clinic during the first cross-section and is extrapolated for the same individuals for the second cross-section. For the third cross-section all individuals were asked about their smoking status during the phone interview. ** — for the purposes of the analysis we excluded vaccinated individuals from the tested subsample of individuals in the fourth cross-section, assumed that they failed to agree to get tested, and used their predicted seropositivity status from our univariate imputation model rather than the actual test results.

**Table S6.** Representativeness of the survey across study cross-sections

| | Interviewed, 2020-05-25 – 2020-06-28 | Interviewed, 2020-10-12 – 2020-12-06 | KOUZh-2018 |
|---|---|---|---|
| Male, % | 41.2 | 42.5 | 40.2 |
| | (40.0–42.4) | (41.4–43.6) | (38.4–41.9) |
| Age, years | 43.0 | 44.1 | 46.7 |
| | (42.7–43.4) | (43.8–44.5) | (46.1–47.3) |
| 18-34, % | 36.3 | 33.4 | 27.9 |
| | (35.1–37.5) | (32.3–34.5) | (26.3–29.5) |
| 35-49, % | 31.8 | 31.9 | 31.7 |
| | (30.7–32.9) | (30.9–33.0) | (30.0–33.3) |
| 50-64, % | 19.9 | 20.6 | 22.8 |
| | (19.0–20.9) | (19.7–21.5) | (21.3–24.3) |
| 65+, % | 11.9 | 14.1 | 17.6 |
| | (11.1–12.7) | (13.3–14.9) | (16.3–19.0) |
| Education | | | |
| Primary / secondary education, % | 10.0 | 10.5 | 12.9 |
| | (9.2–10.7) | (9.8–11.2) | (11.7–14.1) |
| Special secondary education, % | 23.0 | 27.4 | 39.5 |
| | (22.0–24.1) | (26.4–28.4) | (37.7–41.3) |
| Higher education, % | 67.0 | 62.0 | 47.6 |
| | (65.9–68.2) | (60.9–63.1) | (45.8–49.4) |
| Employed, % | 68.3 | 65.0 | 70.2 |
| | (67.1–69.4) | (64.0–66.1) | (68.6–71.8) |
| Current smoker*, % | 20.5 | 31.5 | 31.6 |
| | (18.0–23.1) | (30.5–32.6) | (30.0–33.3) |
| Self-reported health status | | | |
| Very good, % | 19.9 | 16.2 | 7.8 |
| | (18.9–20.9) | (15.4–17.0) | (6.8–8.7) |
| Good, % | 48.9 | 48.6 | 45.3 |
| | (47.7–50.1) | (47.5–49.7) | (43.5–47.1) |
| Satisfactory, % | 28.5 | 32.3 | 39.2 |
| | (27.4–29.6) | (31.2–33.3) | (37.5–41) |
| Bad, % | 2.4 | 2.4 | 7.1 |
| | (2.0–2.8) | (2.0–2.7) | (6.2–8.0) |
| Very bad, % | 0.3 | 0.5 | 0.6 |
| | (0.2–0.5) | (0.4–0.7) | (0.3–0.9) |
| Lives alone, % | 18.7 | 19.1 | 19.7 |
| | (17.7–19.6) | (18.2–19.9) | (18.3–21.1) |
| Has cellphone, % | 100 | 100 | 99.5 |
| | | | (99.3–99.8) |
| N | 6336 | 7595 | 2977 |

95% confidence intervals in parentheses. "Interviewed" means individuals who agreed to participate in the respective cross-section of the phone survey. KOUZh-2018 is the 2016 round of the Comprehensive Monitoring of Living Conditions household survey carried out by the Federal State Statistics Service of Russia. We subset this survey to include only adults in St. Petersburg. We report only complete-case observations in terms of all variables, therefore the number of observations is slightly lower due to listwise deletion. * — current smoking status variable is gathered from the paper-based survey of tested individuals in the clinic during cross-section 1 (N = 949).

**Table S7.** Seroprevalence by cross-section: naïve adjusted for non-response bias or adjusted for non-response and test performance

| test | CMIA Abbott | | | | ELISA Coronapass | | | | ELISA Vector | | | |
|---|---|---|---|---|---|---|---|---|---|---|---|---|
| estimate | naïve | naïve | adjusted | adjusted | naïve | naïve | adjusted | adjusted | naïve | naïve | adjusted | adjusted |
| raking | no | yes | no | yes | no | yes | no | yes | no | yes | no | yes |
| 2020-10-12 – 2020-12-06 | 14.2 (11.5–16.9) | 14.3 (11.6–17.0) | 11.5 (9.0–14.1) | 11.2 (8.2–14.3) | 11.6 (9.5–13.6) | 11.6 (9.5–13.7) | 9.7 (7.7–11.7) | 9.4 (7.0–11.8) | — | — | — | — |
| 2020-07-20 – 2020-08-08 | 21.1 (16.5–25.7) | 19.7 (15.2–24.3) | 16.1 (11.8–20.4) | 14.0 (9.3–18.6) | 16.5 (13.0–20.0) | 15.5 (12.0–18.9) | 13.3 (9.9–16.6) | 11.4 (7.9–15.0) | — | — | — | — |
| 2020-10-12 – 2020-12-06 | 24.9 (22.0–27.9) | 25.4 (22.4–28.4) | 22.0 (19.0–25.1) | 22.0 (18.5–25.6) | 25.2 (22.8–27.7) | 26.4 (23.9–28.9) | 22.9 (20.3–25.5) | 23.8 (20.7–26.9) | 26.9 (24.3–29.4) | 28.1 (25.5–30.6) | 23.9 (21.3–26.4) | 24.6 (21.5–27.7) |
| 2020-02-15 – 2020-04-04 | — | — | — | — | 57.9 (54.7–61.0) | 56.5 (53.4–59.7) | 43.9 (39.7–48.0) | 42.1 (37.5–46.8) | 58.2 (55.1–61.3) | 57.1 (54.0–60.2) | 49.5 (45.7–53.2) | 48.6 (44.2–52.9) |

**Table S8.** Seroprevalence by subgroup, ELISA Coronapass

| | | 2020-05-25 – 2020-06-28 | | 2020-07-20 – 2020-08-08 | | 2020-10-12 – 2020-12-06 | | 2020-02-15 – 2020-04-04 | |
|---|---|---|---|---|---|---|---|---|---|
| | | *N* | seroprevalence | *N* | seroprevalence | *N* | seroprevalence | *N* | seroprevalence |
| | | naïve | | | | | | | |
| Age group | 18-34 | 387 | 13.8 (10.2–17.4) | 172 | 16.4 (10.6–22.2) | 499 | 24.4 (20.4–28.4) | 391 | 51.7 (46.3–57.1) |
| | 35-49 | 341 | 9.2 (6.0–12.5) | 158 | 16.5 (10.4–22.6) | 479 | 25.9 (21.7–30.0) | 428 | 61.5 (56.4–66.6) |
| | 50-64 | 199 | 13.7 (8.6–18.7) | 116 | 20.6 (12.9–28.4) | 267 | 28.1 (22.4–33.8) | 257 | 65.1 (58.6–71.6) |
| | 65+ | 61 | 3.6 (0.0–8.4) | 28 | — | 77 | 16.9 (8.1–25.7) | 64 | 42.5 (29.5–55.5) |
| Female | | 623 | 11.0 (8.4–13.6) | 300 | 16.7 (12.2–21.1) | 874 | 23.9 (20.9–26.9) | 747 | 54.0 (50.1–57.9) |
| Male | | 365 | 12.5 (8.9–16.1) | 174 | 16.2 (10.5–22.0) | 448 | 27.9 (23.5–32.3) | 393 | 65.3 (60–70.5) |
| Higher education | no | 168 | 13.6 (8.2–19.0) | 68 | 12.8 (4.5–21.1) | 261 | 28.3 (22.5–34.1) | 200 | 48.9 (41.4–56.4) |
| | yes | 820 | 11.1 (8.9–13.4) | 406 | 17.1 (13.3–21) | 1061 | 24.5 (21.8–27.2) | 940 | 59.8 (56.3–63.2) |
| Higher income | no | 491 | 11.1 (8.2–14.0) | 221 | 13.8 (9.0–18.5) | — | — | — | — |
| | yes | 497 | 12.0 (9.0–15.0) | 253 | 18.9 (13.8–24.0) | — | — | — | — |
| Respondent lives alone | no | 803 | 12.5 (10.1–14.8) | 372 | 18.4 (14.3–22.6) | 1094 | 26 (23.3–28.8) | 951 | 59.8 (56.3–63.2) |
| | yes | 185 | 7.6 (3.6–11.6) | 102 | 9.6 (3.6–15.6) | 228 | 21.5 (15.8–27.1) | 189 | 48.3 (40.6–56.0) |
| History of COVID-19 testing | no | 760 | 8.4 (6.4–10.5) | 357 | 11.6 (8.1–15.0) | 726 | 16.2 (13.4–19.0) | 319 | 35.4 (29.8–41.0) |
| | yes | 228 | 21.9 (16.3–27.6) | 117 | 31.6 (22.6–40.5) | 596 | 36.3 (32.2–40.4) | 821 | 66.6 (63–70.2) |
| Current smoker | no | — | — | — | — | 982 | 27.2 (24.3–30.2) | — | — |
| | yes | — | — | — | — | 340 | 19.5 (15.1–23.9) | — | — |
| History of illnesses in the past 3 months | no | 547 | 5.2 (3.2–7.1) | 266 | 9.8 (6.1–13.5) | 407 | 11.8 (8.5–15.0) | 319 | 35.4 (29.8–41.0) |
| | yes | 441 | 19.5 (15.6–23.4) | 208 | 25.1 (18.9–31.3) | 915 | 31.2 (28.1–34.4) | 821 | 66.6 (63.0–70.2) |
| Started to wash hands more often since pandemic | no | 279 | 16.4 (11.8–20.9) | 128 | 24.6 (16.7–32.5) | 389 | 26.0 (21.4–30.6) | — | — |
| | yes | 709 | 9.7 (7.4–11.9) | 346 | 13.5 (9.7–17.3) | 933 | 24.9 (22.0–27.9) | — | — |
| | | adjusted for non-response bias and test characteristics | | | | | | | |
| Age group | 18-34 | 387 | 12.1 (8.7–15.5) | 172 | 14.0 (8.8–19.2) | 499 | 23.2 (19.4–27.0) | 391 | 41.5 (36.1–47.0) |
| | 35-49 | 341 | 8.0 (5.1–10.8) | 158 | 15.5 (9.7–21.3) | 479 | 24.1 (20.2–28.1) | 428 | 48.1 (42.4–53.8) |
| | 50-64 | 199 | 11.6 (7.1–16.0) | 116 | 15.5 (8.7–22.3) | 267 | 25.0 (19.8–30.2) | 257 | 50.4 (43.1–57.6) |
| | 65+ | 61 | 3.3 (0.0–7.7) | 28.0 | — | 77 | 15.9 (7.8–24) | 64 | 29.5 (18.4–40.5) |
| Female | | 623 | 9.3 (7.0–11.7) | 300 | 14.2 (10.2–18.2) | 874 | 22.1 (19.1–25.1) | 747 | 37.9 (33.4–42.4) |
| Male | | 365 | 10.2 (7.1–13.4) | 174 | 11.9 (7.1–16.8) | 448 | 23.9 (19.9–27.9) | 393 | 52.0 (46.0–57.9) |
| Higher education | no | 168 | 10.5 (6.2–14.7) | 68 | 8.8 (2.7–14.8) | 261 | 24.1 (19.0–29.2) | 200 | 37.3 (30.2–44.4) |
| | yes | 820 | 9.4 (7.3–11.4) | 406 | 15.4 (11.6–19.2) | 1061 | 22.1 (19.6–24.7) | 940 | 47.5 (43.2–51.8) |
| Higher income | no | 491 | 9.2 (6.6–11.8) | 221 | 11.1 (7.1–15.2) | — | — | — | — |
| | yes | 497 | 10.4 (7.6–13.2) | 253 | 16.1 (11.2–20.9) | — | — | — | — |
| Respondent lives alone | no | 803 | 10.6 (8.3–12.8) | 372 | 14.6 (10.7–18.4) | 1094 | 23.8 (21.0–26.6) | 951 | 45.5 (41.0–49.9) |
| | yes | 185 | 5.9 (2.6–9.2) | 102 | 7.4 (2.6–12.2) | 228 | 18.7 (13.8–23.7) | 189 | 36.9 (29.8–44.0) |
| History of COVID-19 testing | no | 760 | 7.7 (5.6–9.7) | 357 | 10.2 (6.8–13.5) | 726 | 15.2 (12.3–18.1) | 319 | 34.3 (28.8–39.9) |
| | yes | 228 | 20.9 (15.4–26.3) | 117 | 30.2 (21.3–39.1) | 596 | 36 (31.8–40.3) | 821 | 66.5 (62.7–70.3) |
| Current smoker | no | — | — | — | — | 982 | 24.8 (21.9–27.8) | — | — |
| | yes | — | — | — | — | 340 | 18.5 (14.3–22.8) | — | — |
| History of illnesses in the past 3 months | no | 547 | 5.4 (3.3–7.5) | 266 | 9.0 (5.4–12.7) | 407 | 11.7 (8.3–15.2) | 319 | 39.6 (34.8–44.5) |
| | yes | 441 | 18.9 (14.9–22.8) | 208 | 22.3 (16.3–28.2) | 915 | 31.5 (28.1–34.9) | 821 | 49.1 (45.7–52.6) |
| Started to wash hands more often since pandemic | no | 279 | 12.7 (8.8–16.5) | 128 | 19.2 (12.6–25.8) | 389 | 24.0 (19.6–28.4) | — | — |
| | yes | 709 | 8.2 (6.1–10.3) | 346 | 10.2 (6.9–13.5) | 933 | 22.2 (19.3–25.1) | — | — |

**Figure S1.** Naïve and adjusted seroprevalence by study cross-section and week (ELISA Coronapass)

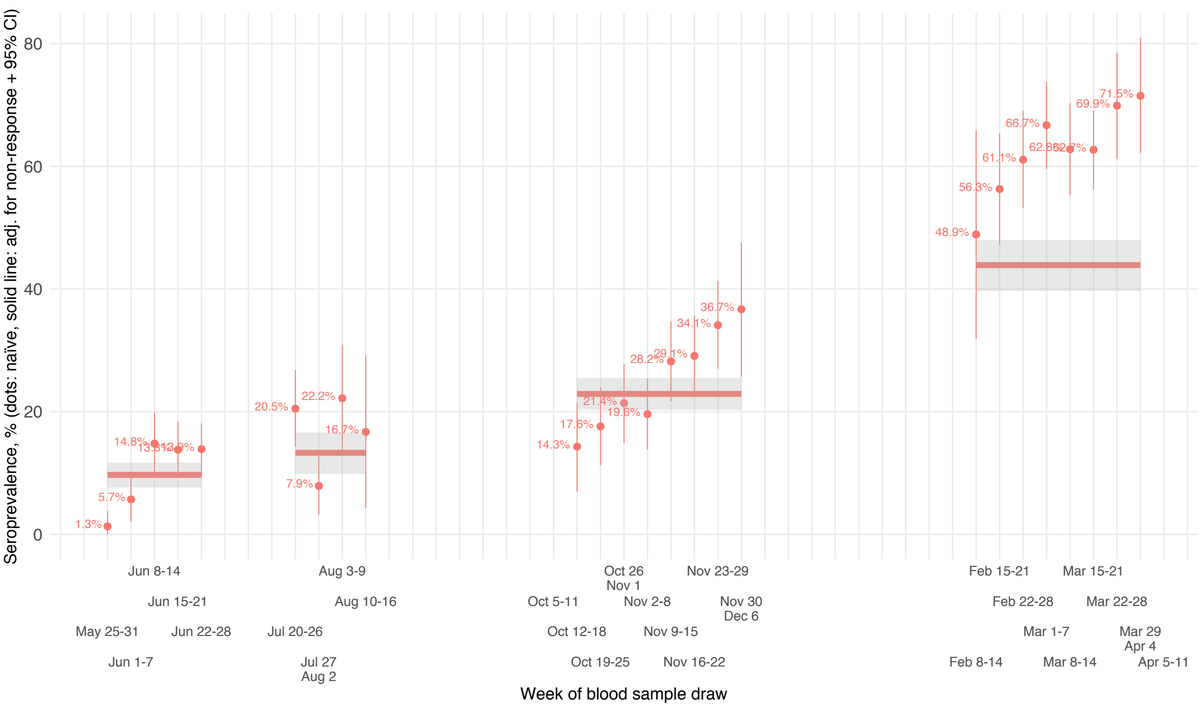

Supplement: S1 Appendix — (PDF) [file pone.0266945.s001.pdf]
